# Intronic *TP53* Polymorphisms Are Associated with Increased *Δ133TP53* Transcript, Immune Infiltration and Cancer Risk

**DOI:** 10.3390/cancers12092472

**Published:** 2020-09-01

**Authors:** Ramona A. Eiholzer, Sunali Mehta, Marina Kazantseva, Catherine J. Drummond, Cushla McKinney, Katie Young, David Slater, Brianna C. Morten, Kelly A. Avery-Kiejda, Annette Lasham, Nicholas Fleming, Helen R. Morrin, Karen Reader, Janice A. Royds, Michael Landmann, Simone Petrich, Roger Reddel, Lily Huschtscha, Ahmad Taha, Noelyn A. Hung, Tania L. Slatter, Antony W. Braithwaite

**Affiliations:** 1 Department of Pathology, Dunedin School of Medicine, University of Otago, Dunedin 9016, New Zealand; ramona.eiholzer@otago.ac.nz (R.A.E.); sunali.mehta@otago.ac.nz (S.M.); marina.kazantseva@otago.ac.nz (M.K.); cath.drummond@otago.ac.nz (C.J.D.); cushla.mckinney@otago.ac.nz (C.M.); katie.young@otago.ac.nz (K.Y.); nicholas.fleming@otago.ac.nz (N.F.); karen.reader@otago.ac.nz (K.R.); janice.royds@otago.ac.nz (J.A.R.); noelyn.hung@otago.ac.nz (N.A.H.); tania.slatter@otago.ac.nz (T.L.S.); 2 Maurice Wilkins Centre for Molecular Biodiscovery, Dunedin 9016, New Zealand; 3 Priority Research Centre for Cancer Research, Innovation and Translation and the Hunter Cancer Research Alliance, School of Biomedical Sciences and Pharmacy, Faculty of Health and Medicine, University of Newcastle, New South Wales 2305, Australia; David.w.slater22@gmail.com (D.S.); Brianna.Morten@uon.edu.au (B.C.M.); kelly.kiejda@newcastle.edu.au (K.A.A.-K.); 4 Hunter Medical Research Institute, New South Wales 2305, Australia; 5 Department of Molecular Medicine and Pathology, University of Auckland, Auckland 1023, New Zealand; a.lasham@auckland.ac.nz; 6 Department of Pathology and Biomedical Science, University of Otago, Christchurch 8140, New Zealand; helen.morrin@otago.ac.nz; 7 Department of Surgical Sciences, Dunedin School of Medicine, University of Otago, Dunedin 9054, New Zealand; michael.landmann@otago.ac.nz (M.L.); simone.petrich@southerndhb.govt.nz (S.P.); 8 Children’s Medical Research Institute, University of Sydney, Westmead, New South Wales 2145, Australia; RReddel@cmri.org.au (R.R.); lhuschtscha@gmail.com (L.H.); 9 Department of Neurosurgery, Southern District Heath Board, Dunedin 9016, New Zealand; ahmad.taha@southerndhb.govt.nz; 10 Malaghan Institute of Medical Research, Wellington 6242, New Zealand

**Keywords:** single nucleotide polymorphism, *TP53*, glioblastoma, prostate cancer, Δ133p53, rs1042522, rs9895829 and rs2909430

## Abstract

**Simple Summary:**

We investigated the influence of genetic variants, called single nucleotide polymorphisms (SNP) in the *TP53* tumour suppressor gene, on cancer risk, clinical features and *TP53* isoform levels. These SNPs were significantly over-represented in cohorts of mixed cancers versus controls, suggesting they confer increased cancer risk. Heterozygosity at rs1042522(GC) and either of the two SNPs rs9895829(TC) and rs2909430(AG) confer up to a 5-fold greater risk of developing cancer. The SNP combinations were associated with high *Δ133TP53* and *TP53β* messenger RNA levels, elevated infiltrating immune cells and shorter patient survival for glioblastoma and prostate cancer. The data suggest that ∆133p53β protein levels are increased by the SNPs resulting in increased inflammation which contributes to more aggressive cancers.

**Abstract:**

We investigated the influence of selected *TP53* SNPs in exon 4 and intron 4 on cancer risk, clinicopathological features and expression of *TP53* isoforms. The intron 4 SNPs were significantly over-represented in cohorts of mixed cancers compared to three ethnically matched controls, suggesting they confer increased cancer risk. Further analysis showed that heterozygosity at rs1042522(GC) and either of the two intronic SNPs rs9895829(TC) and rs2909430(AG) confer a 2.34–5.35-fold greater risk of developing cancer. These SNP combinations were found to be associated with shorter patient survival for glioblastoma and prostate cancer. Additionally, these SNPs were associated with tumor-promoting inflammation as evidenced by high levels of infiltrating immune cells and expression of the *Δ133TP53* and *TP53β* transcripts. We propose that these SNP combinations allow increased expression of the Δ133p53 isoforms to promote the recruitment of immune cells that create an immunosuppressive environment leading to cancer progression.

## 1. Introduction

p53 is a powerful tumours suppressor [1] with defects in the p53 pathway being an almost universal hallmark of human cancers [2,3]. Mice deleted for the *TP53* gene are highly tumour prone [1] and in Li–Fraumeni syndrome (LFS) where mutations in the *TP53* gene are inherited, multiple tumour types occur [4]. Despite this, identifying *TP53* mutations alone has limited power in predicting patient outcome [5,6,7,8].

Apart from point mutations and protein-coding transcripts, the *TP53* gene contains over 200 single nucleotide polymorphisms (SNPs). One of the most well studied *TP53* polymorphisms is rs1042522 (G > C) that changes the amino acid at codon 72 from Arginine to Proline (P72R > P). This change in amino acid leads to a structural alteration in the protein, which alters the function of p53 [9,10,11]. For example, p53R72 (R72) induces apoptosis more effectively than p53P72 (P72) [12], whereas P72 appears to induce a greater G_1_ arrest [9,13]. Furthermore, P72 is more efficient than R72 in activating several p53-dependent DNA repair and other genes, and cells with P72 have a significantly higher DNA repair capacity and reduced genome instability [13,14].

The rs1042522 SNP has been studied for its contribution to disease risk for most of the common cancer types including gliomas [15], cervical [16,17], lung [18], prostate [19], bladder [20], stomach [21], breast [22] and colorectal cancers [23]. However, the findings are inconsistent [15,16,17,18,19,20,21,22,23]. This may be attributed to differences in sample size, anatomic location of the tissue, ethnicities, haplotype effects or genotyping methods used among various studies [15,16,17,18,19,20,21,22,23]. However, there are examples of the rs1042522 SNP affecting diseases associated with inflammation [9,24,25,26], such as type II diabetes [27,28], rheumatoid arthritis [29,30] and ulcerative colitis (UC) [31].

Other than rs1042522 SNP, common haplotypes found in the P2 promoter in intron 4 [32] are associated with increased lung cancer risk and poor prognosis [33]. Another study demonstrated that there are 8 known *TP53* polymorphisms in 11 common haplotypes that are present within the P2 promoter [32]. Using promoter constructs, this study identified 2/11 haplotypes markedly increased the baseline expression of the *∆133TP53* family of isoforms [34].

*∆133TP53* is one of 4 families of isoforms encoded in the *TP53* locus. These isoforms differ at the N-terminus by use of alternative promoter usage, alternative initiation of translation, and by alternate splicing at the C-terminus (rviewed in detail [5,6,35]). Several studies have now shown that aberrant expression of some p53 isoforms contribute to diseases, including cancer [5,6,35]. Elevated transcript levels of the *∆133TP53* isoforms are associated with poor patient outcomes in breast [36,37], prostate [38] and colorectal cancers [39] and the *∆133TP53β* isoform level is associated with increased immune cell infiltration in glioblastoma [35] and prostate cancer [38]. Other evidence suggests that ∆133p53 isoforms can antagonise canonical full-length p53 tumour suppressor activity and have multiple intrinsic tumour-promoting capacities [40].

Given this background, we investigated the influence of zygosity in addition to haplotype variation between the R72P SNP (rs1042522) and two P2 promoter SNPs in intron 4 on cancer risk. We hypothesized that the combination of these SNPs may affect transcription of the *Δ133TP53* isoforms. We report that the combination of R72P SNP and intron 4 SNPs was associated with increased cancer risk; affected transcript levels of *Δ133TP53* isoforms, and was associated with increased immune infiltrate and poor survival in glioblastoma and prostate cancer patients.

## 2. Results

### 2.1. Intronic SNPs rs9895829 and rs2909430 Are Associated with Increased Cancer Risk

**Figure 1:**
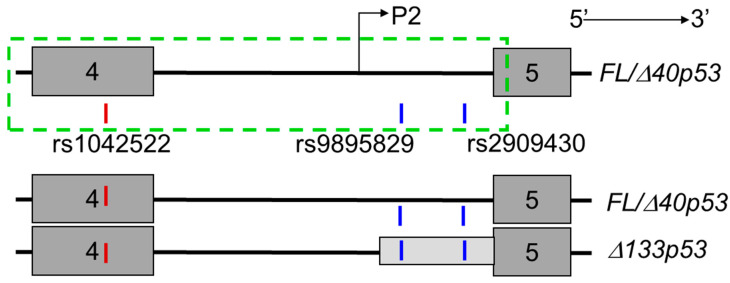
Schematic illustrating the location of rs1042522, rs9895829 and rs2909430 within the *TP53* gene spanning exons 4–5. The dark grey boxes indicate the exons, the light grey box indicates the untranslated region (UTR) of the *Δ133TP53* transcripts. The black line connecting the exons (dark grey boxes) indicates the intronic region (intron 4). P2–P2 promoter, and the dotted green box indicates the genomic region representing the P2 promoter. The exonic SNP is shown as a red vertical line (rs1042522) and intronic single nucleotide polymorphisms (SNPs) are shown as blue vertical lines (rs9895829 and rs2909430).

Five SNPs in *TP53* (rs1042522, rs35850753, rs1794287, rs9895829 and rs2909430) were genotyped using blood or normal associated tissue from individuals that had cancer [33,34]. Consistent with the literature our sequencing data showed rs9895829/rs35850753 and rs2909430/rs1794287 are in high linkage disequilibrium and form haplotype blocks [33,34]. The SNP data for rs9895829 and rs2909430 are reflective of the SNPs at rs35850753 and rs1794287 respectively. Thus, to avoid redundant comparison, we selected rs9895829 and rs2909430 as tagging SNPs for rs35850753 and rs1794287 respectively for further analyses (Figure 1), along with rs14042522. The study cohort comprised individuals with glioblastoma (GBM, *n* = 89), prostate (PCa, *n* = 122) and breast (BrCa, *n* = 389) cancers respectively. Ethnically matched control cohorts were derived from the 1000 genomes [41] (Control Cohort 1), the Caucasian case-control cohort from Mechanic et al. [33] (Control Cohort 2) and the Dunedin Multi-Disciplinary Health and Development Study [42] (Control Cohort 3).

The frequency of rs1042522 was similar in the study population and control populations (Table 1). However, the frequencies of the other two SNPs (rs9895829 and rs2909430) were significantly different between the study and control populations (Table 1). These results suggest that these two intronic SNPs are associated with an increased risk of cancer.

Consistent, with previous studies [9,24,25,26], cloning and sequencing the region spanning exons 4–5 in several tumour types showed that the minor allele (MA) of the intronic SNPs was primarily found in combination with the “C” allele for rs1042522 (Figure S1). We, therefore, tested each intron 4 SNP in combination with rs1042522 (rs22) for association with cancer risk. To do this we compared the study cohort to control cohort 1 since it was the only one with the intronic SNP data. Individuals that are heterozygous for rs1042522(CG/GC) hereafter are referred to as rs22(GC), for rs9895829(TC/CT) as rs29(TC) and rs2939430(AG/GA) as rs30 (AG). Results from the study cohort compared to control cohort 1 indicated that individuals with rs22(GC)+rs30(AG) SNP combination had a 2.34-fold increased risk of cancer while those with rs22(GC)+rs29(TC) SNP combination had a 5.35-fold increased risk of cancer (Table 2). None of the individuals in the control cohorts that were homozygous for rs22(GG), were heterozygous for rs29(TC), but this combination was present in nearly half the study cohort (Table 2). Similarly, only one individual that was homozygous for rs22(GG) was heterozygous for rs30(AG) in control cohort 1 compared to 182 individuals with the SNP combination in the study cohort (Table 2). These results suggest, that individuals that are heterozygous at rs22(GC) and are heterozygous for either of the two intronic SNPs rs29(TC) and rs30(AG) have a significantly higher likelihood of having cancer.

### 2.2. Association of TP53 Polymorphisms with Patient Survival

**Figure 2:**
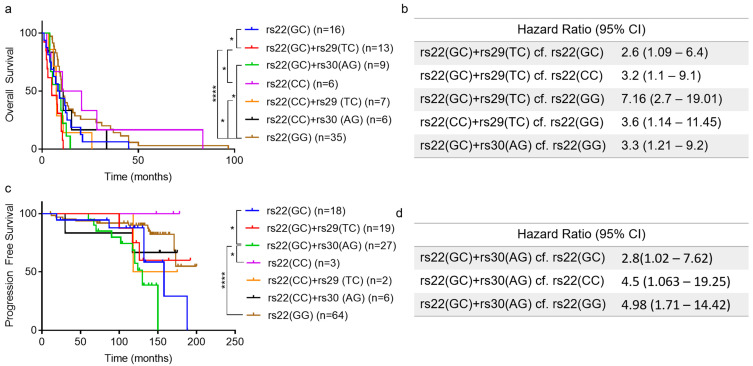
Intronic SNPs (rs29 and rs30) are associated with shorter survival of glioblastoma and prostate cancer patients. (**a**) Overall Survival of glioblastoma patients. (**b**) Hazard ratio (95% CI, significant *p*-value, <0.05) for groups with significant differences in overall survival (GBM patients). (**c**) Progression free survival of prostate cancer patients. (**d**) Hazard ratio (95% CI, significant *p*-value, <0.05) for groups with significant progression-free survival (PCa patients). (**a**,**c**). Kaplan–Meier analysis, significance determined using the log-rank test (*—significant *p*-value, * < 0.05, **** < 0.0001).

To determine if the combinations of *TP53* SNPs were associated with cancer patient outcomes we performed survival analyses on the GBM and PCa cohorts after stratification for SNP combinations. Results for GBM patients show that individuals with the rs22(GC)+rs29(TC) SNP combination had significantly shorter overall survival than individuals with rs22(GC), rs22(CC) and rs22(GG) combinations (Hazard ratio: 2.6–7.16, Figure 2a,b). Similarly, individuals with the rs22(CC)+rs29(TC) or rs22(GC)+rs30(AG) SNP combinations had significantly shorter overall survival than individuals with the rs22(GG) SNP alone (Hazard Ratio: 3.3–3.6, Figure 2a,b).

Next, we assessed the association of these *TP53* SNPs on PCa patient outcome. Results from progression-free survival (PFS) analysis identified individuals with the rs22(GC)+rs30(AG) SNP combination as having significantly shorter PFS compared to individuals with rs22(GC), rs22(CC) and rs22(GG) (Hazard ratio: 2.8–4.98, Figure 2c,d). Interestingly, only the rs22(GC)+rs30(AG) SNP combination was associated with survival outcome for PCa patients, while in GBMs both rs22(GC)+rs29(TC) and rs22(GC)+rs30(AG) SNP combinations were associated with patient survival. Taken together, these results suggest that certain SNP combinations may influence cancer outcomes for individuals with GBM and PCa.

### 2.3. Association of TP53 Polymorphisms with Clinical Parameters of Cancer Patients

GBM can be stratified by telomere maintenance mechanisms. They can be telomerase (TEL) positive or positive for the Alternative Lengthening of Telomeres (ALT) mechanism [43,44]. In addition, GBMs can be further stratified based on the content of CD163+ macrophages (Mɸ) [45,46]. Both parameters affect patient survival with those that are TEL positive and have a high Mɸ content (TELM) have the poorest outcome [43,45,46]. We, therefore, assessed the influence of the three *TP53* SNP combinations on these parameters. Our analysis showed that individuals with rs22(GC)+rs29(TC) were more likely to have TELM GBMs compared to rs22(GC) and rs22(CC) (Table S1). Similarly, individuals with rs22(GC)+rs30(AG) were more likely to have TELM GBMs compared to rs22(CC) (Table S2).

**Figure 3:**
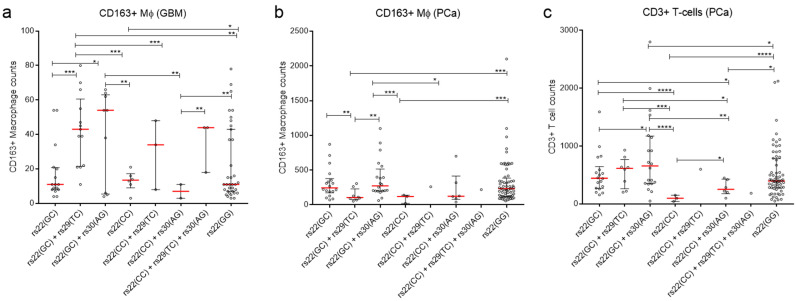
Intronic SNPs (rs29 and rs30) influences the amount of CD163+ Mɸ in GBM and PCa patients and CD3+ T cells in PCa patients. (**a**) CD163+ Mɸ count in GBMs. (**b**) CD163+ Mɸ count in PCa. (**c**) CD3+ T cell count in PCa. (**a**–**c**) Dot plot of CD163+ Mɸ or CD3+ T cells. Each dot represents the respective immune cell count from individual tumours. Red line—median with whiskers—interquartile range. Significance was determined using the Welch’s t-test. *—*p* value. * < 0.05, ** < 0.01, *** < 0.001 and **** < 0.0001, respectively.

We also looked for an association between genotypes and CD163+ Mɸ content. Results show that tumours from patients with rs22(GC)+rs29(TC) genotype had significantly more CD163+ Mɸ (median 3.19–6.14 fold) compared to rs22(CG), rs22(CC), rs22(GG) and rs22(CC)+rs30(AG) genotypes (Figure 3a and Table S3). Similarly, tumours from patients with rs22(GC)+rs30(AG) genotype had significantly more Mɸ (median 4–7.71 fold) compared to rs22(GC), rs22(CC), rs22(CC)+rs30(AG) genotypes (Figure 3a and Table S3). Taken together, individuals with rs22(GC)+rs29(TC) and rs22(GC)+rs30(AG) genotypes are more likely to have TELM GBMs and therefore poorer outcomes.

We then assessed the association of these SNPs with common clinical parameters of PCa. Our results showed that none of these genotypes was significantly associated with prostate-specific antigen (PSA) levels, whilst individuals with rs22(CC) genotype were significantly less likely to be associated with a Gleason score of ≥7 and a CAPRA score of >3 compared to individuals with rs22(GC) and rs22(GC)+rs30(AG) genotypes (Table S2).

The presence of immune cells that promote an immunosuppressive environment within PCa has been associated with advanced PCa [47,48]. Consistent with this, we demonstrated CD3+ T cells can predict poor PCa outcomes with 79% accuracy and CD163+ Mɸ cells with 68% accuracy [38]. Thus, we assessed the influence of the three *TP53* SNP combinations on immune cell content. Results show that tumours from patients with the SNP combination rs22(GC)+rs30(AG) had significantly more CD163+ Mɸ (median 2.28–2.56 fold) compared to rs22(GC)+rs29(TC) and rs22(CC) (Figure 3b and Table S3). Individuals also with rs22(GC) and rs22(GG) had tumours that had 2-fold elevated median levels of CD163+ Mɸ counts compared to rs(GC)+rs29(TC), which was also significant (Figure 3b and Table S3). However, there was no significant difference between the median CD163+ Mɸ counts in the tumours of patients with rs22(GC)+rs30(AG) compared to those with rs22(GC), and of rs22(GC)+rs29(TC) compared to the rs22(CC) genotype (Figure 3b and Table S3).

Next, we examined the effect of the different *TP53* SNP combinations on the amount of CD3+ T cells in PCa. Results showed that patients with SNP combination rs22(GC)+rs30(AG) had significantly elevated CD3+ T cells compared to patients with other SNP combinations (Figure 3c and Table S3). Similarly, individuals with SNP combination rs22(GC)+rs29(TC) had significantly elevated CD3+ T cells (Figure 3c and Table S3). Finally, individuals with rs22(GC) and rs22(GG) had significantly elevated median levels of CD3+ T cells compared to rs22(CC) (Figure 3c and Table S3). Thus, similar to the GBM cohort, SNP combinations associated with poor PCa patient outcomes were also associated with a higher content of inflammatory cells.

### 2.4. Association of TP53 Polymorphisms with Δ133TP53 and TP53β Isoform Expression in Cancers

**Figure 4:**
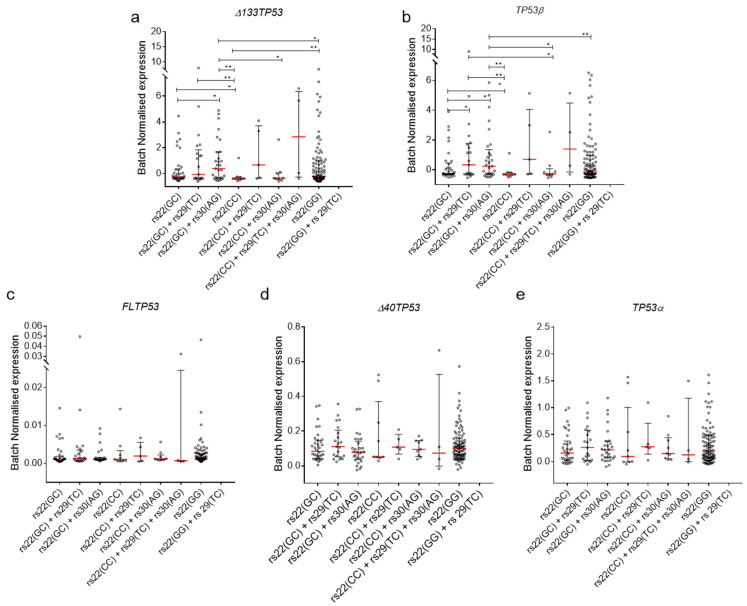
Intronic SNPs (rs29 and rs30) are associated with elevated mRNA levels of *Δ133TP53* and *TP53β* in GBM and PCa. (**a**) *Δ133TP53*, (**b**) *TP53β*, (**c**) *FLTP53*, (**d**) *Δ40TP53* and (**e**) *TP53α* mRNA expression from GBM and PCa. (**a**–**e**) Dot plot of batch normalised mRNA expression of *TP53* isoforms. Red line—median with whiskers—interquartile range. Significance was determined using Welch’s t-test. *—*p* value. * < 0.05, ** < 0.01.

High levels of the *Δ133TP53β* transcript are associated with an inflammatory phenotype [38,49] and poor outcome [38,39]. To test for an association of the SNP combinations present in the P2 promoter with expression data of the *TP53* isoforms, we stratified the GBM and PCa by SNP combinations and published isoform expression data [38,39,49]. Results showed that individuals with rs22(GC)+rs30(AG) SNP combination had tumours with significantly elevated median levels of *Δ133TP53* and *TP53β* mRNA compared to rs22(GC), rs22(CC), and rs22(CC)+rs30(AG) (Figure 4a,b). Interestingly, these individuals also had significantly elevated levels of *Δ133TP53* mRNA alone compared to those with rs22(GG) (Figure 4a). Additionally, individuals with rs22(GC)+rs29(TC) SNP combinations had tumours with significantly elevated median levels of *Δ133TP53* and *TP53β* mRNA compared to rs22(CC) alone (Figure 4a,b). Finally, none of the three *TP53* SNPs was associated with altered mRNA levels of other *TP53* transcripts (Figure 4c–e). Taken together, these results suggest that the presence of the minor allele for either of the two intronic SNPs in combination with heterozygous rs22 SNP is associated with elevated levels of the *Δ133TP53* family and the *TP53β* splice variant in these tumour types.

### 2.5. Association of TP53 Polymorphisms with Δ133TP53 and TP53β Isoform Expression in Normal Cells

**Figure 5:**
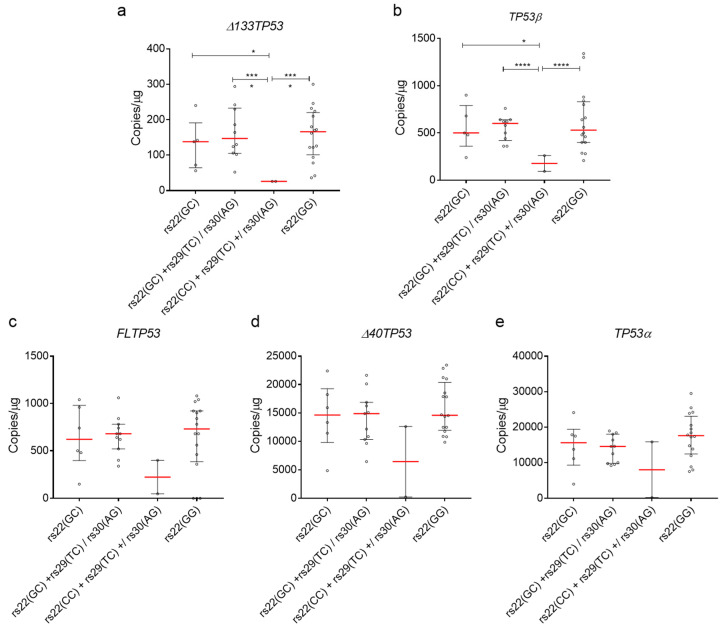
Intronic SNPs (rs29 and rs30) are associated with increased mRNA levels of *Δ133TP53* and *TP53β* in breast fibroblast cell lines. (**a**) *Δ133TP53*, (**b**) *TP53β*, (**c**) *FLTP53*, (**d**) *Δ40TP53* and (**e**) *TP53α* mRNA expression from breast fibroblasts. (**a**–**e**) Dot plot of batch normalised mRNA expression of *TP53* isoforms. Red line—median and whiskers—interquartile range. Significance was determined using Welch’s t-test. *—*p*-value. * < 0.05, *** < 0.001 and **** < 0.0001, respectively.

To test if the combinations of SNPs are associated with basal expression of *Δ133TP53* and *TP53β* mRNAs as a potential mechanism of cancer predisposition as suggested by Bellini et al. [34], we measured the expression of all *TP53* transcripts in 33 breast fibroblast cell lines, stratified by SNP combinations. We combined the rs22(GC)+rs30(AG) SNP with rs22(GC)+rs29(TC) and rs22(CC)+rs29(TC) with rs22(CC)+rs29(TC)+rs30(AG) as there was only 1 cell line with the former combination and 2 cell lines with the latter combination. Thus, we stratified the isoform expression into 4 groups as follows rs22(GC), rs22(GC)+rs29(TC)/rs30(AG), rs22(CC)+rs29(TC)+/rs30(AG) and rs22(GG). Consistent with the results from the tumours, cell lines with rs22(GC)+rs29(TC)/rs30(AG) combination had the highest median expression of *Δ133TP53* and *TP53β* mRNAs (Figure 5a,b). Interestingly, both rs22(GC) and rs22(GG) also had a significantly higher median expression for *Δ133TP53* and *TP53β* mRNAs compared to cell lines with rs22(CC)+rs29(TC)/rs30(AG) (Figure 5a,b). The basal expression of other *TP53* transcripts was not significantly different (Figure 5c–e). Results from these cell lines provide further evidence that there is an association with these SNP combinations and increased mRNA expression of *Δ133TP53* and *TP53β* isoforms.

## 3. Discussion

We investigated the influence of *TP53* SNPs in exon 4/intron 4 on cancer risk, clinicopathological parameters, patient survival and the mRNA expression of the *TP53* isoforms. The minor alleles of the intron 4 SNPs were significantly over-represented in the cohorts of mixed cancers we studied compared to three control cohorts, suggesting that they confer increased cancer risk. Further analysis showed that heterozygosity at rs22(GC) and at either of the two intronic SNPs rs29(TC) and rs30(AG) conferred a greater risk of developing cancer. Our analysis found individuals with rs22(GC)+rs30(AG) were more likely to have aggressive PCa characterised by increased immune infiltration (CD163+ Mɸ and CD3+ T cells) and elevated levels *Δ133TP53* and *TP53β* mRNA compared to rs22(CC), rs22(GC) and rs22(GG) individuals. Our previous studies in GBM [49], PCa [38] and CRC [39] have identified a correlation coefficient of >0.95 between *Δ133TP53* and *TP53β*, leading to the inference that the *Δ133TP53β* is the most likely *Δ133TP53* isoform being expressed. We have recently shown that PCa with elevated levels *Δ133TP53β* isoform and increased immune cell infiltration are associated with poor outcome [38]. Consistent with these observations we found that individuals with rs22(GC)+rs30(AG) had significantly shorter progression-free PCa survival and were more likely to have aggressive TELM GBMs. In GBMs, we also found that individuals with the SNP combination rs22(GC)+rs29(TC) were more likely to have aggressive GBMs characterised by increased immune infiltration (CD163+ Mɸ) and elevated levels *Δ133TP53β* isoform. Consistent with our observations that TELM tumours are associated with poor outcomes [49], we observed that individuals with GBM and rs22(GC)+rs29(TC) SNP combination were associated with the shortest overall survival.

A previous study showed that the rs22C+rs29T+rs30A haplotype had a poor outcome in lung cancer patients in African Americans but not in Caucasians [33], although there was no association with *TP53* isoform expression done. In our studies, compound heterozygosity at rs22(GC) and intron 4 SNP are predictive of cancer predisposition and prognosis in Caucasians. These SNP combinations are also associated with elevated *Δ133TP53β* transcription.

Given this context, we propose that the *TP53* SNPs investigated here allow increased transcription of the *Δ133TP53* mRNAs, as suggested by Bellini et al. [34] using in vitro P2 promoter assays. Our data would also suggest that the SNPs may influence 3′ splicing to favour the *TP53β* variant. This then leads to increased Δ133p53β, which in turn increases the levels of chemokines and cytokines to provoke inflammation involving immune cells that create an immunosuppressive environment and cancer progression.

Our model predicts that the SNP combinations above provide a DNA template structure conducive to increased transcription from the *TP53* P2 promoter. The data from Bellini et al. suggested that the presence of rs1794287 in the P2 promoter can alter binding affinity for several transcription factors [34]. Although there are no experimental data confirming this hypothesis, in silico modelling of the effect of the three SNPs (rs22, rs29 and rs30) on the structure of the P2 promoter [50] also suggest that each of these SNPs alters DNA conformation and interaction with a number of transcription factors (determined using TFBind [51], Figure S2). For example, the prediction is that heterozygosity at rs30 allows the binding of 13 unique transcription factors in addition to 6 common transcription factors. Similarly, heterozygosity at rs29 allows the binding of 8 unique transcription factors plus 9 common transcription factors. It is also possible that heterozygosity observed at rs22, rs29 and rs30 might act cooperatively with each other, thus playing an important role in determining the final activity of the P2 promoter. Pre-mRNA splicing is frequently coupled to transcription by RNA polymerase II (RNAPII) [52]. Accumulation of transcription factors at the P2 promoter can alter the rate of elongation by Pol II, facilitating the recruitment of splicing factors that favour retention of intron 9β. This would provide a possible explanation for increased *Δ133TP53β* mRNA expression in individuals heterozygous for rs22 in combination with rs29 or rs30 compared to other SNP combinations. Increased Δ133p53β would in turn promote a tumour-favouring microenvironment, resulting in aggressive disease. Characterization of differentially bound transcription factors to these sequences remains to be explored experimentally.

As the presence of “C” or “G” allele at rs1042522 influences P2 promoter activity and *Δ133TP53β* expression [34], our results suggest that C allele dampens expression of *Δ133TP53β*. However, this can be rescued by two mechanisms: (i) selecting for heterozygosity at rs1042522 in combination with haplotypes of the C allele that contain the minor allele at either rs29 or rs30, or (ii) selecting for G allele homozygosity at rs22. As rs22(CC) is at a very low frequency in Caucasians, selecting for genotype combinations that increase Δ133p53 expression has presumably been advantageous during Caucasian evolution. This may be due to the pro-inflammatory properties of the Δ133p53 isoforms enabling increased adaptability to rapidly changing environmental conditions.

Our studies highlight the potential importance of SNP combinations in contributing to disease risk. However, our conclusions need to be tempered due to the low numbers of samples we have in our cohorts, which is exacerbated by the various stratifications we have done. Nonetheless, identification of individuals not only at risk of developing the disease but also having knowledge of disease severity or sub-type is clearly important. Thus, confirmation of our findings in multiple and larger patient cohorts is required.

## 4. Materials and Methods

### 4.1. Control Cohorts

Three control cohorts were used in this study. All three cohorts were sourced from different geographical locations. Control cohort 1 consisted of 599 subjects from the 1000 genomes [41] database consisting of Utah Residents (CEU), Colombians from Medellin, Columbia (CLM), Finnish from Finland (FIN), British from England and Scotland (GBR), Iberian population from Spain (IBS) and Toscani in Italia (TSI). Control cohort 2 consisted of 335 Caucasian controls from the study conducted in greater Baltimore, Maryland in the United States of America, details of which are described in Mechanic et al. [33]. Control cohort 3 consisted of 668 New Zealand/European individuals born in Dunedin, New Zealand in 1972/1973, details of which are described in Hancox et al. [42].

### 4.2. Cancer Cohort

The cancer cohort was comprised of glioblastomas (GBM), prostate cancers (PCa), and 2 breast cancer (BrCa) cohorts. The GBM consisted of 89 individuals previously studied [49]. Ethical approval (LRS/10/09/037/AM05 and MEC/08/02/061/AM13) was obtained in New Zealand and all procedures followed institutional guidelines. The PCa cohort consisted of 122 individuals which was obtained from the Cancer Society Tissue Bank (16/STH/92 Christchurch, NZ) and described in detail previously [38]. Ethical approval (LRS/10/09/037/AM05) was obtained. BrCa cohort 1 consisted of 75 individuals from Dunedin and Christchurch (New Zealand Health & Disability Ethics Committee Reference LRS/10/09/035 [53,54]). BrCa cohort 2 consisted of 314 breast cancer cases from Australia, which was obtained from the Australian Breast Cancer Tissue Bank (ABCTB) with ethical approval from the Hunter New England Human Research Ethics Committee (Approval number: 09/05/20/5.01). Informed patient consent was obtained for all cohorts.

### 4.3. Cell Culture

The human breast fibroblast cell lines (Fre lines) were obtained from Professor Roger Reddel and derived from post breast reduction surgery. There were 35 Fre lines included in this study and they are Fre 105, Fre 76, Fre 94, Fre 111, Fre 103, Fre 120, Fre 97, Fre 106, Fre 107, Fre 108, Fre 131, Fre 146, Fre 141, Fre 119, Fre 135, Fre 142, Fre 71, Fre 98, Fre 99, Fre 143, Fre 74, Fre 88, Fre 96, Fre 101, Fre 102, Fre 129, Fre 134, Fre 92, Fre 110, Fre 137, Fre 138, Fre 121, Fre 133, Fre 139 and Fre 140. The Adventist HealthCare Ltd. Human Research Ethics Committee, Wahroonga NSW Australia, granted Ethics for the use of these cells. Cells were incubated in a humidified atmosphere containing 5% CO_2_ at 37 °C and cultured in DMEM (Life Technologies, Carlsbad, CA, USA) supplemented with 10% FBS (Sigma-Aldrich, St. Louis, Mo, USA).

### 4.4. Cloning Experiments

The P2 promoter fragments of the *TP53* gene were obtained from human genomic DNA from patients heterozygous for the rs22 and the rs29 or rs30 SNPs using 5′-TCCTCTGACTGCTCTTTTCACCCATC-3′ (sense primer) and 5′-CCACACGCAAATTTCCTTCCA-3′ (antisense primer), generating a product of 1383 nt (nucleotide)from –1169 nt to +214 nt (relative to the ATG initiation codon of *∆133TP53*, Figure S1). Each 50 μL of PCR reaction mixture contained 5 μL of 10 × GXL Buffer (PrimeSTAR, Takara Bio, Kusatsu, Shiga, Japan), 0.5 μL of dNTP Mixture, 0.5 μL of forward primer (10 μM), 0.5 μL of reverse primer (10 μM), 36 μL of water, 100 ng of genomic DNA, and 0.1 μL of Platinum Taq DNA Polymerase (Life Technologies, Carlsbad, CA, USA). The reaction conditions were as follows: 95 °C for 3 min, 35 cycles of 95 °C for 30 s; 56 °C for 30s and 72 °C for 2 min, final step of 72 °C for 10 min. Amplicons were cloned into pCR8/GW/TOPO vector (Invitrogen, Waltham, MA, USA) as per the manufacturer’s instructions followed by Sanger sequencing in both directions.

### 4.5. Genotyping

Genotyping for each cancer cohort is as described here. DNA was extracted from the blood of individuals with GBM, BrCa cohort 1 and from normally associated tissue of individuals with PCa. DNA was extracted from the Fre lines for genotyping. For all tumour cohorts studied in Dunedin and the Fre lines, PCR was used to amplify the DNA region between exons 4–9 of *TP53*. The primer sequences used were those published [55]. For amplification of intron 4, the following primers were also used: 5′-AGTTTACCCACTTAATGTGTG-3′; 5′-CAGGAGATGGAGGCTGCAGTG-3′; 5′-CAAGGCAGGCAGATCACCTG-3′ (sense primers) and 5′-CTATAGGTGTGCACCACCATG-3′; 5′-CCTCCTGCAACCCACTAG-3′; 5′-CCACAGCTGCACAGGGCAGG-3′ (antisense primers). Purified PCR products were subjected to Sanger sequencing to identify SNPs.

For BrCa cohort 2, genomic DNA (2.5ng) from the blood of breast cancer patients at diagnosis was genotyped using a 96.96 genotyping integrated fluidics circuit (IFC) with custom SNP-type assays on the Juno^TM^ system (Fluidigm, South San Francisco, CA, USA) with quantitation on the Biomark^TM^ (Fluidigm, South San Francisco, USA), according to the manufacturers’ instructions. The data were analysed using the Fluidigm SNP Genotyping Analysis software version 4.5.1 (South San Francisco, CA, USA), to obtain genotype calls.

### 4.6. Preparation of RNA, cDNA and ddPCR for Analysis of p53 Isoforms

RNA (1µg) extracted from the 33 cell lines was DNase I treated (Thermo Fisher Scientific, Waltham, MA, USA) and then reverse-transcribed using qScript cDNA SuperMix (Quanta Biosciences, Beverly, MA, USA), according to manufacturer’s instructions. Primers designed for specific *TP53* transcript subclasses (*FL/Δ40TP53_T1* referred to as FLp53, *FL/Δ40TP53_T2* referred to as Δ40p53 and *Δ133TP53*, *TP53α* and *TP53β*) were used from previous studies [56,57]. Absolute transcript abundance was measured with EvaGreen SuperMix using the Bio-Rad QX200 ddPCR System (Bio-Rad, Hercules, CA, USA) and converted to copies/µg RNA, as described previously [56,57].

### 4.7. Statistical Analyses

A Chi-square test was used to assess significant differences in the distribution of SNPs between control and cancer cohorts. Two-sided Fisher’s exact test was used to assess the significant differences and Odds ratios were calculated using the Baptista–Pike method. Differences between survival curves were tested using Kaplan–Meier analysis followed by a two-sided log-rank test. Comparisons between groups were done using unpaired one-tailed t-test with Welch’s correction. All Statistical analyses were performed using GraphPad Prism software version 7.03.

## 5. Conclusions

Our study found that zygosity of rs1042522 and two intron 4 SNPs rs9895829 and rs2909430 are associated with increased cancer risk. Heterozygous combinations of rs1042522 with either rs9895829 or rs2909430 also affected the transcript levels of *Δ133TP53* and *TP53β* isoforms. Heterozygous combinations of these SNPs were associated with poor patient outcome and increased immune cell infiltration in glioblastoma and prostate cancers. In conclusion, our studies highlight how genomic variations within the *TP53* locus profoundly affect the risk of developing cancer as well as cancer progression.

## Figures and Tables

**Table 1:** Comparison of rs1042522, rs9895829 and rs2909430 frequencies in the study cohort-1 (cancer) and control cohorts.

**Genotype** **(rs1042522)**	**Study Cohort-1**	**Control Cohort 1**	**χ** **^2^** **-Test** ***p*** **-Value**	**Control Cohort 2**	**χ** **^2^** **-Test** ***p*** **-Value**	**Control Cohort 3**	**χ** **^2^** **-Test** ***p*** **-Value**
GG (REF)	326	302	0.27	193	0.4223	373	
CG/GC	226	253		122		247	0.79
CC (MA)	48	44		20		48	
**Genotype** **(rs9895829)**	**Study Cohort-1**	**Control Cohort 1**	**χ** **^2^** **-test** ***p*** **-value**	**Control Cohort 2**	**χ** **^2^** **-test** ***p*** **-value**	**Control Cohort 3**	**χ** **^2^** **-test** ***p*** **-value**
TT (REF)	291	535	<0.0001 (****)	304	<0.0001 (****)		
TC/CT	302	62		21		NA	
CC (MA)	7	2		0			
**Genotype** **(rs2909430)**	**Study Cohort-1**	**Control Cohort 1**	**χ** **^2^** **-test** ***p-*** **value**	**Control Cohort 2**	**χ** **^2^** **-test** ***p-*** **value**	**Control Cohort 3**	**χ** **^2^** **-test** ***p-*** **value**
AA (REF)	227	441	<0.0001 (****)	254	<0.0001 (****)		
AG/GA	370	150		68		NA	
GG (MA)	3	8		5			

* χ2—Chi-square test. REF—reference sequence; MA—minor allele, NA—data not available.

**Table 2:** Association of *TP53* haplotypes with cancer.

Genotype	Control Cohort 1	Study Cohort	OR (95% CI), Fisher’s Exact Test *p*-Value		
			rs22(CC)+rs30(AG)	rs22(CC)+rs30(GG)	rs22(CC)+rs30(AA)
rs22(CC)+rs30(AA)	17	18	1.34 (0.53–3.13), 0.82		
rs22(CC)+rs30(AG)	19	27		0.26 (0.07–1.1), 0.09	
rs22(CC)+rs30(GG)	8	3			0.35 (0.09–1.6), 0.18
			rs22(GG)+rs30(AG)	rs22(GG)+rs30(GG)	rs22(GG)+rs30(AA)
rs22(GG)+rs30(AA)	301	144	380.4 (66.24–3826), 0.0001		
rs22(GG)+rs30(AG)	1	182		ND	
rs22(GG)+rs30(GG)	0	0			ND
			rs22(GC)+rs30(AG)	rs22(GC)+rs30(GG)	rs22(GC)+rs30(AA)
rs22(GC)+rs30(AA)	123	65	2.34 (1.6–3.4), 0.0001		
rs22(GC)+rs30(AG)	130	161		ND	
rs22(GC)+rs30(GG)	0	0			ND
			rs22(CC)+rs29(TC)	rs22(CC)+rs29(CC)	rs22(CC)+rs29(TT)
rs22(CC)+rs29(TT)	31	25	2.3 (0.95–5.9), 0.07		
rs22(CC)+rs29(TC)	11	21		0.54(0.075–3.76), 0.6	
rs22(CC)+rs29(CC)	2	2			1.24 (0.18–8.3), 0.99
			rs22(GG)+rs29(TC)	rs22(GG)+rs29(CC)	rs22(GG)+rs29(TT)
rs22(GG)+rs29(TT)	302	172	∞ (75.89–∞), 0.0001		
rs22(GG)+rs29(TC)	0	154		ND	
rs22(GG)+rs29(CC)	0	0			ND
			rs22(GC)+rs29(TC)	rs22(GC)+rs29(CC)	rs22(GC)+rs29(TT)
rs22(GC)+rs29(TT)	202	94	5.35 (3.53–8.01), 0.0001		
rs22(GC)+rs29(TC)	51	127		∞ (0.57–∞), 0.32	
rs22(GC)+rs29(CC)	0	5			∞ (3.1–∞), 0.0036

* ND—Not Determined, ∞—infinity, OR—Odds Ratio, CI—Confidence Interval.

## Supplementary Materials

The following are available online at https://www.mdpi.com/2072-6694/12/9/2472/s1, Figure S1: The presence of rs29 and rs30 SNPs is associated with the “C” allele for rs22, Figure S2: Presence of rs29 and rs30 SNPs alters DNA conformation and transcription factor binding, Table S1: Association of *TP53* polymorphisms with Telomerase maintenance status (TMM status) in GBMs, Table S2: Association of *TP53* polymorphisms with PSA levels (ng/µl), Gleason Score and CAPRA Score in PCa, Table S3: Presence of intronic SNPs (rs29 and rs30) influences the amount of CD163+ Mɸ in GBM and PCa patients and CD3+ T cells in PCa patients.
