# Supplementary material for: Intronic TP53 Polymorphisms Are Associated with Increased Δ133TP53 Transcript, Immune Infiltration and Cancer Risk
Source: Cancers (Basel). 2020 Sep 1;12(9):2472. doi: 10.3390/cancers12092472 (PMC7563340; doi:10.3390/cancers12092472)
Supplement: Supplementary file 1 [file cancers-12-02472-s001.pdf]

cancers

MDPI

Supplementary material

Intronic *TP53* Polymorphisms are Associated with Increased *Δ133TP53* Transcript, Immune Infiltration and Cancer Risk

Ramona A. Eiholzer, Sunali Mehta, Marina Kazantseva, Catherine J Drummond, Cushla McKinney, Katie Young, David Slater, Brianna C. Morten, Kelly A. Avery-Kiejda, Annette Lasham, Nicholas Fleming, Helen R. Morrin, Karen Reader, Janice A. Royds, Michael Landmann, Simone Petrich, Roger Reddel, Lily Huschtscha, Ahmad Taha, Noelyn A. Hung, Tania L. Slatter and Antony W. Braithwaite

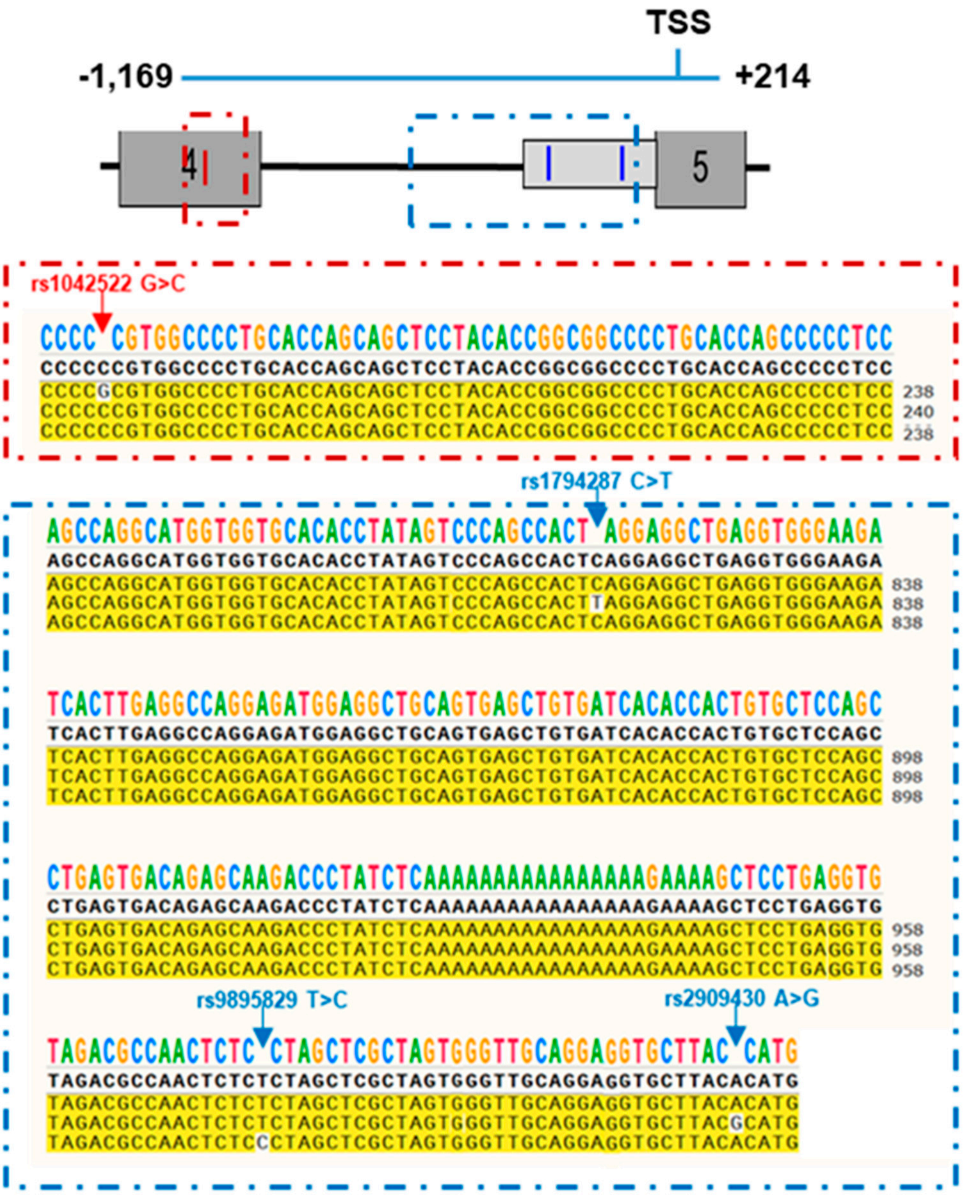

Figure S1. The presence of rs29 and rs30 SNPs is associated with the "C" allele for rs22. Schematic representation of the amplified fragment of the TP53 P2 promoter ranging from -1,169 nt to +214 nt (relative to the ATG initiation codon of Δ133TP53) cloned into TOPO vector. Shown are representative results obtained with Sanger sequencing of the P2 fragments containing either "G" or "C" allele for the rs22 (boxed in red) showing the presence of rs29 or rs30 SNPs on the "C" allele for rs22 (boxed in blue). TSS, transcription start site.

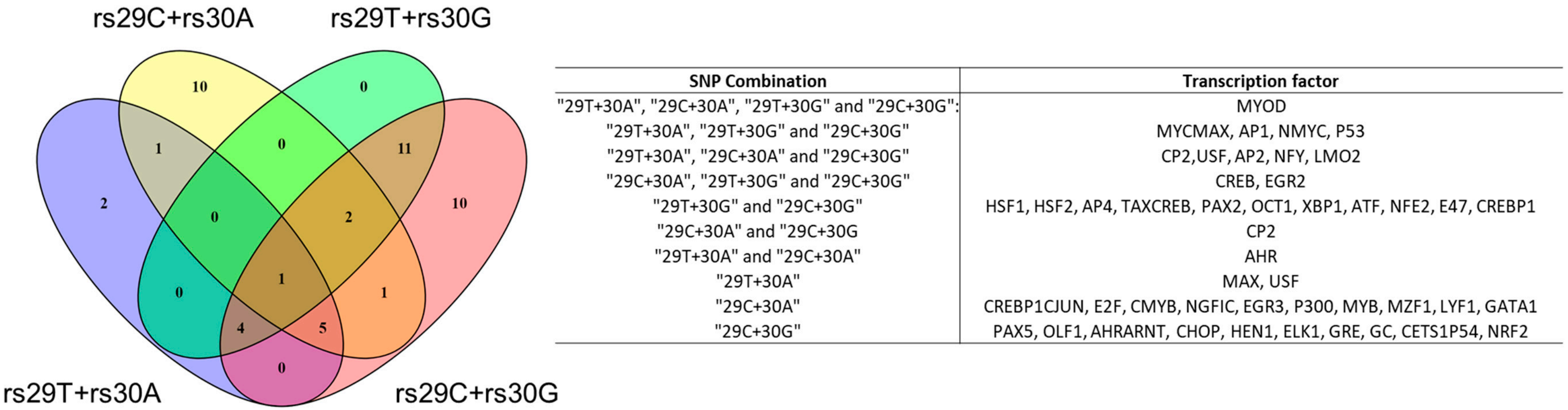

SNP Combination	Transcription factor
"29T+30A", "29C+30A", "29T+30G" and "29C+30G":	MYOD
"29T+30A", "29T+30G" and "29C+30G"	MYCMAX, AP1, NMYC, P53
"29T+30A", "29C+30A" and "29C+30G"	CP2,USF, AP2, NFY, LMO2
"29C+30A", "29T+30G" and "29C+30G"	CREB, EGR2
"29T+30G" and "29C+30G"	HSF1, HSF2, AP4, TAXCREB, PAX2, OCT1, XBP1, ATF, NFE2, E47, CREBP1
"29C+30A" and "29C+30G	CP2
"29T+30A" and "29C+30A"	AHR
"29T+30A"	MAX, USF
"29C+30A"	CREBP1CJUN, E2F, CMYB, NGFIC, EGR3, P300, MYB, MZF1, LYF1, GATA1
"29C+30G"	PAX5, OLF1, AHRARNT, CHOP, HEN1, ELK1, GRE, GC, CETS1P54, NRF2

Figure S2. Presence of rs29 and rs30 SNPs alters DNA conformation and transcription factor binding. Shows a Venn diagram with the number of unique and common transcription factors able to bind DNA with the tested combinations for rs29 and rs30. The table shows details of the transcription factors that bind DNA with various tested combinations for rs29 and rs30. Changes in DNA sequence was determined after modelling using RNA Structure [1]. DNA sequences used for Transcription factor binding for each of the combinations is as follows: rs29T and 30A – 5′-ATCTCTAGCTTCGCTAGTGGGTTGC-3′ and 5′- CTTACACATGTTTGT-3′; rs29C+rs30T – 5′-AGACGCCAACTCTCCCTAGCTCGCTAGTGGGTTGC-3′, rs29T+rs30G – 5′- AGGAGGTGCTTACGCATGTTTGT-3′ and rs29C+rs30G – 5′-TCCCTAAGCTCGCTAGTGGGTTGCAGGAGGTGCTTACGCATGTTTGT-3′. Alphabets highlighted in red either the rs29 or rs30 nucleotide. Transcription factors binding each of these sequences was determined using TFBind [2]. The Venn diagram was drawn using Venny [3].

Table 1. Association of TP53 polymorphisms with Telomerase maintenance status (TMM status) in GBMs.

TMM Status	Genotype 1	Genotype 2	OR (95% CI), Fisher's exact test *p* value		
	rs22(GC)	rs22(GC) + rs29(TC)	TEL	TELM	ALT
ALT	3	1	0.9 (0.09–15.22), 0.99		
TEL	10	3		10 (1.58–47.93), 0.017	
TELM	3	9			9 (0.86–126.8), 0.11
	rs22(GC) + rs29(TC)	rs22(CC)	TEL	TELM	ALT
ALT	1	1	1.67(0.06–37.27), >0.99		
TEL	3	5		0 (0–0.49), 0.009	
TELM	9	0			0 (0–2), 0.18
	rs22(GC) + rs30(AG)	rs22(CC)	TEL	TELM	ALT
ALT	1	1	2.5(0.09–55.71), >0.99		
TEL	2	5		0 (0–0.67), 0.021	
TELM	6	0			0 (0–3), 0.25
	rs22(GC) + rs29(TC)	rs22(GC) + rs30(AG)	TEL	TELM	ALT
ALT	1	1	0.67(0.02–18.51), >0.99		
TEL	3	2		1(0.16–6.95), >0.99	
TELM	9	6			0.67(0.032–14.61), >0.99
	rs22(GC) + rs29(TC)	rs22(GG)	TEL	TELM	ALT
ALT	1	10	0.47(0.03–3.62), >0.99		
TEL	3	14		0.26 (0.067–1.26), 0.09	
TELM	9	11			0.12(0.01–0.96), 0.0458
	rs22(GC) + rs29(TC)	rs22(CC) + rs29(TC)	TEL	TELM	ALT
ALT	1	0	∞ (0.05–∞), >0.99		
TEL	3	2		0.67(0.10–5.01), >0.99	
TELM	9	4			∞ (0.04–∞), >0.99
	rs22(GC) + rs29(TC)	rs22(CC) + rs30(AG)	TEL	TELM	ALT
ALT	1	1	0.67(0.02–18.51), >0.99		
TEL	3	2		0.33 (0.04–3.06), 0.55	
TELM	9	2			0.22(0.01–6.2), 0.42
	rs22(GC) + rs30(AG)	rs22(GG)	TEL	TELM	ALT
ALT	1	10	0.7(0.04–6.75), 0.64		

TEL	2	14		0.26(0.04–1.5), 0.13	
TELM	6	11			0.18(0.01–1.76), 0.19
	rs22(GC) + rs30(AG)	rs22(CC) + rs29(TC)	TEL	TELM	ALT
ALT	1	0	∞ (0.07–∞), >0.99		
TEL	2	2		0.67(0.08–5.8), >0.99	
TELM	6	4			∞ (0.06–∞), >0.99
	rs22(GC) + rs30(AG)	rs22(CC) + rs30(AG)	TEL	TELM	ALT
ALT	1	1	1 (0.03–27.69), >0.99		
TEL	2	2		0.33 (0.03–3.56), 0.55	
TELM	6	2			0.33(0.015–9.29), >0.99
	rs22(GC)	rs22(GG)	TEL	TELM	ALT
ALT	3	10	0.42 (0.10–2.04), 0.3		
TEL	10	14		2.6(0.56–10.32), 0.29	
TELM	3	11			1.1(0.22–5.57), >0.99
	rs22(GC)	rs22(CC)	TEL	TELM	ALT
ALT	3	1	1.5(0.17–22.97), >0.99		
TEL	10	5		0(0–3.05), 0.52	
TELM	3	0			0(0–12), >0.99
	rs22(GG)	rs22(CC)	TEL	TELM	ALT
ALT	10	1	3.57(0.49–45.75), 0.37		
TEL	14	5		0(0–1.16), 0.13	
TELM	11	0			0(0–9), >0.99

Table S2. Association of TP53 polymorphisms with PSA levels (ng/µl), Gleason Score and CAPRA Score in PCa. ∞–Infinity.

**GENOTYPE**	**PSA**		**Odds Ratio (95% CI),**	**Gleason Score**		**Odds Ratio (95% CI),**	**CAPRA Score**		**Odds Ratio (95% CI),**
	**<10**	**≥10**	**Fishers Exact Test *p* value**	**<7**	**≥7**	**Fishers Exact Test *p* value**	**≤3**	**>3**	**Fishers Exact Test *p* value**
rs22(GC)	10	8	0(0–1.78)	4	14	0(0–0.46)	5	13	0(0–0.6)
rs22(CC)	3	0	0.25	3	0	0.026	3	0	0.042
rs22(GC) + rs30(AG)	17	3	0(0–10.45)	6	14	0 (0 to 0.64)	5	15	0 (0–0.52)
rs22(CC)	3	0	>0.99	3	0	0.047	3	0	0.031
rs22(GC)	10	8	0.17(0.01–1.72)	4	14	0.28(0.04–1.48)	5	13	0.23(0.05–1.35)
rs22(GC) + rs29(TC)	7	1	0.19	4	4	0.19	5	3	0.18

rs22(GC)	10	8	0.22(0.05–1.11)	4	14	0.67(0.18–2.61)	5	13	1.15(0.26–4.97)
rs22(GC) + rs30(AG)	17	3	0.07	6	14	0.72	13	15	>0.99
rs22(GC)	10	8	0.61(0.21–1.9)	4	14	0.3(0.10–0.95)	5	13	0.38(0.14–1.11)
rs22(GG)	43	21	0.40	31	33	0.06	32	32	0.11
rs22(GC)	10	8	1.25(0.05–25.87)	4	14	∞ (0.11–∞)	5	13	0.38 (0.01–8.67)
rs22(CC)+rs29 (TC)	1	1	>0.99	0	2	>0.99	1	1	0.52
rs22(GC)	10	8	0.93(0.19–4.73)	4	14	0.38(0.05–2.09)	5	13	0.15(0.02–0.96)
rs22(CC)+rs30 (AG)	4	3	>0.99	3	4	0.35	5	2	0.07
rs22(GC) + rs29(TC)	7	1	1.25(0.15–18.11)	4	4	2.33(0.51–10.93)	5	3	5(0.74–22.96)
rs22(GC) + rs30(AG)	17	3	>0.99	6	14	0.4	5	15	0.09
rs22(GC) + rs29(TC)	7	1	0 (0–24)	4	4	0(0–2.06)	5	3	0(0–3.83)
rs22(CC)	3	0	>0.99	3	0	0.23	3	0	0.49
rs22(GC) + rs29(TC)	7	1	7 (0.18–147.6)	4	4	∞ (0.31–∞)	5	3	1.67(0.06–37.27)
rs22(CC)+rs29 (TC)	1	1	0.37	0	2	0.46	1	1	>0.99
rs22(GC) + rs29(TC)	7	1	5.25(0.54–76.22)	4	4	1.33(0.14–8.12)	5	3	0.67(0.09–4.63)
rs22(CC)+rs30 (AG)	4	3	0.28	3	4	>0.99	5	2	>0.99
rs22(GC) + rs29(TC)	7	1	3.41(0.53–40.15)	4	4	1.06(0.28–3.91)	5	3	1.67(0.38–6.67)
rs22(GG)	43	21	0.42	31	33	>0.99	32	32	0.71
rs22(GC) + rs30(AG)	17	3	5.7(0.22–112.3)	6	14	∞ (0.17–∞)	5	15	0.33(0.01–7.52)
rs22(CC)+rs29 (TC)	1	1	0.33	0	2	>0.99	1	1	0.48
rs22(GC) + rs30(AG)	17	3	4.25(0.72–22.68)	6	14	0.57(0.11–2.88)	5	15	0.13(0.02–0.84)
rs22(CC)+rs30 (AG)	4	3	0.28	3	4	0.65	5	2	0.06
rs22(GC) + rs30(AG)	17	3	2.76(0.78–9.6)	6	14	0.45(0.152–1.36)	5	15	0.33(0.12–1.05)
rs22(GG)	43	21	0.16	31	33	0.19	32	32	0.07
rs22(CC)	3	0	∞ (0.39–∞)	3	0	∞ (0.86–∞)	3	0	∞ (0.81–∞)
rs22(GG)	43	21	0.54	31	33	0.23	32	32	0.24

Table S3. Presence of intronic SNPs (rs29 and rs30) influences the amount of CD163+ Mϕ in GBM and PCa patients and CD3+ T cells in PCa patients.

Genotypes	GBM CD163+ Macrophages			PCa CD163+ Macrophages			PCa CD3+ T cells		
	**Median (25th–75th Percentile)**	**Fold Change**	**Welch's t-test *p* value**	**Median (25th–75th Percentile)**	**Fold Change**	**Welch's t-test *p* value**	**Median (25th–75th Percentile)**	**Fold Change**	**Welch's t-test *p* value**
rs22(GC) + rs29(TC)<br>rs22(GC)	43(21.5–60.5)<br>11(8–20.75)	3.91	0.0008	104.5(75.25–229.3)<br>245.5(173–378.3)	0.43	0.005	615(263–766.8)<br>444(268.5–644.8)	1.39	0.4042
rs22(GC) + rs29(TC)<br>rs22(CC)	43(21.5–60.5)<br>13.5(9–17.25)	3.19	0.0001	104.5(75.25–229.3)<br>120(21–140)	0.87	0.1924	615(263–766.8)<br>98(42–150)	6.28	0.0007
rs22(GC) + rs29(TC)<br>rs22(CC) + rs30(AG)	43(21.5–60.5)<br>7(3–11)	6.14	0.0007	104.5(75.25–229.3)<br>124(79–415)	0.84	0.2073	615(263–766.8)<br>252.5(175–428)	2.44	0.0139
rs22(GC) + rs29(TC)<br>rs22(GG)	43(21.5–60.5)<br>11(7–43)	3.91	0.0039	104.5(75.25–229.3)<br>232.5(123.3–386)	0.45	0.0005	615(263–766.8)<br>397.5(280.3–781.5)	1.55	0.4395
rs22(GC) + rs30(AG)<br>rs22(GC)	54(5.5–63)<br>11(8–20.75)	4.91	0.0244	274(200–517)<br>245.5(173–378.3)	1.12	0.2153	656(350–1174)<br>444(268.5–644.8)	1.48	0.0389
rs22(GC) + rs30(AG)<br>rs22(CC)	54(5.5–63)<br>13.5(9–17.25)	4.00	0.0099	274(200–517)<br>120(21–140)	2.28	0.0006	656(350–1174)<br>98(42–150)	6.69	<0.0001
rs22(GC) + rs30(AG)<br>rs22(CC) + rs30(AG)	54(5.5–63)<br>7(3–11)	7.71	0.005	274(200–517)<br>124(79–415)	2.21	0.1289	656(350–1174)<br>252.5(175–428)	2.60	0.0012
rs22(GC)<br>rs22(CC)	11(8–20.75)<br>13.5(9–17.25)	0.81	0.0169	245.5(173–378.3)<br>120(21–140)	2.05	0.0024	444(268.5–644.8)<br>98(42–150)	4.53	<0.0001
rs22(CC) + rs30(AG)<br>rs22(CC) + rs29(TC) + rs30(AG)	7(3–11)<br>44(18–44)	0.16	0.0338	124(79–415)<br>220(220–220)	0.56	ND	252.5(175–428)<br>185(185–185)	1.36	ND
rs22(CC) + rs30(AG)<br>rs22(GG)	7(3–11)<br>11(7–43)	0.64	0.0266	124(79–415)<br>232.5(123.3–386)	0.53	0.2115	252.5(175–428)<br>397.5(280.3–781.5)	0.64	0.0018
rs22(GC) + rs29(TC)<br>rs22(CC) + rs29(TC)	43(21.5–60.5)<br>34(8–48)	1.26	0.196	104.5(75.25–229.3)<br>261(261–261)	0.40	ND	615(263–766.8)<br>600(600–600)	1.03	ND
rs22(GC) + rs29(TC)<br>rs22(GC) + rs30(AG)	43(21.5–60.5)<br>54(5.5–63)	0.80	0.3645	104.5(75.25–229.3)<br>274(200–517)	0.38	0.0012	615(263–766.8)<br>656(350–1174)	0.94	0.0594
rs22(GC) + rs29(TC)	43(21.5–60.5)	0.98	0.2513	104.5(75.25–229.3)	0.48	ND	615(263–766.8)	3.32	ND

rs22(CC) + rs29(TC) + rs30(AG)	44(18–44)			220(220–220)			185(185–185)		
rs22(GC) + rs30(AG)<br>rs22(CC) + rs29(TC)	54(5.5–63)<br>34(8–48)	1.59	0.2808	274(200–517)<br>261(261–261)	1.05	ND	656(350–1174)<br>600(600–600)	1.09	ND
rs22(GC) + rs30(AG)<br>rs22(CC) + rs29(TC) + rs30(AG)	54(5.5–63)<br>44(18–44)	1.23	0.3824	274(200–517)<br>220(220–220)	1.25	ND	656(350–1174)<br>185(185–185)	3.55	ND
rs22(GC) + rs30(AG)<br>rs22(GG)	54(5.5–63)<br>11(7–43)	4.91	0.0606	274(200–517)<br>232.5(123.3–386)	1.18	0.2498	656(350–1174)<br>397.5(280.3–781.5)	1.65	0.0365
rs22(GC)<br>rs22(CC) + rs29(TC)	11(8–20.75)<br>34(8–48)	0.32	0.2003	245.5(173–378.3)<br>261(261–261)	0.94	ND	444(268.5–644.8)<br>600(600–600)	0.74	ND
rs22(GC)<br>rs22(CC) + rs30(AG)	11(8–20.75)<br>7(3–11)	1.57	0.0709	245.5(173–378.3)<br>124(79–415)	1.98	0.2544	444(268.5–644.8)<br>252.5(175–428)	1.76	0.0119
rs22(GC)<br>rs22(CC) + rs29(TC) + rs30(AG)	11(8–20.75)<br>44(18–44)	0.25	0.0804	245.5(173–378.3)<br>220(220–220)	1.12	ND	444(268.5–644.8)<br>185(185–185)	2.40	ND
rs22(GC)<br>rs22(GG)	11(8–20.75)<br>11(7–43)	1.00	0.166	245.5(173–378.3)<br>232.5(123.3–386)	1.06	0.4154	444(268.5–644.8)<br>397.5(280.3–781.5)	1.12	0.4442
rs22(CC)<br>rs22(CC) + rs29(TC)	13.5(9–17.25)<br>34(8–48)	0.40	0.141	120(21–140)<br>261(261–261)	0.46	ND	98(42–150)<br>600(600–600)	0.16	ND
rs22(CC)<br>rs22(CC) + rs30(AG)	13.5(9–17.25)<br>7(3–11)	1.93	0.1693	120(21–140)<br>124(79–415)	0.97	0.1204	98(42–150)<br>252.5(175–428)	0.39	0.0118
rs22(CC)<br>rs22(CC) + rs29(TC) + rs30(AG)	13.5(9–17.25)<br>44(18–44)	0.30	0.0569	120(21–140)<br>220(220–220)	0.55	ND	98(42–150)<br>185(185–185)	0.53	ND
rs22(CC)<br>rs22(GG)	13.5(9–17.25)<br>11(7–43)	1.23	0.1751	120(21–140)<br>232.5(123.3–386)	0.52	0.001	98(42–150)<br>397.5(280.3–781.5)	0.25	<0.0001
rs22(CC) + rs29(TC)<br>rs22(CC) + rs30(AG)	34(8–48)<br>7(3–11)	4.86	0.0908	261(261–261)<br>124(79–415)	2.10	ND	600(600–600)<br>252.5(175–428)	2.38	ND
rs22(CC) + rs29(TC)<br>rs22(CC) + rs29(TC) + rs30(AG)	34(8–48)<br>44(18–44)	0.77	0.3672	261(261–261)<br>220(220–220)	1.19	ND	600(600–600)<br>185(185–185)	3.24	ND

rs22(CC) + rs29(TC)<br>rs22(GG)	34(8–48)<br>11(7–43)	3.09	0.3061	261(261–261)<br>232.5(123.3–386)	1.13	ND	600(600–600)<br>397.5(280.3–781.5)	1.51	ND
rs22(CC) + rs29(TC) + rs30(AG)<br>rs22(GG)	44(18–44)<br>11(7–43)	4.00	0.1422	220(220–220)<br>232.5(123.3–386)	0.95	ND	185(185–185)<br>397.5(280.3–781.5)	0.47	ND

*ND – not determined.

References

1. Mathews, D.H. Rna secondary structure analysis using rnastructure. *Curr. Protoc. Bioinform.* **2014**, *46*, 12–16.
2. Tsunoda, T.; Takagi, T. Estimating transcription factor bindability on DNA. *Bioinformatics* **1999**, *15*, 622–630.
3. Oliveros, J.C. (2007) VENNY. An interactive tool for comparing lists with Venn Diagrams. Available online: https://bioinfogp.cnb.csic.es/tools/venny/index.html (accessed on 14 April 2020).
